# ABC transporters of *Fasciola hepatica* as putative drug targets

**DOI:** 10.1186/1471-2210-11-S2-A36

**Published:** 2011-09-05

**Authors:** Ali El-Kasaby, Thomas Stockner, Oliver Kudlacek

**Affiliations:** 1 Institute of Pharmacology, Center of Physiology and Pharmacology, Medical University of Vienna, 1090 Vienna, Austria

## Background

The liver fluke *Fasciola hepatica* is one of the most important parasites affecting animal health all over the world, causing the so called liver fluke disease (fascioliosis). Although infections of humans are rather rare in most western countries, several million people worldwide are infected by this trematode. Beside its threat to humans the infection of animal stock leads to large financial losses. As vaccinations against this parasite are not available yet, anthelmintic drugs, like triclabendazole, are the treatment of choice. During the last decades more and more flukes resistant to these drugs have been found. One possible mechanism for these resistances seems to be the expression of so called ABC transporters. Inhibitors of P-glycoprotein (ABCB1) can change the status of flukes from resistant to susceptible. Up to date little is known about proteins expressed by the fluke. In addition almost no information on the fluke’s genome is available. We therefore try to identify and characterize yet unknown proteins of the fluke to investigate their potentency as putative new drug targets.

## Methods

Starting from a previously published sequence of an ABC transporter of *Fasciola hepatica* we used RACE (rapid amplification of cDNA ends) to generate a full length ABC transporter. Heterologous expression of the protein allows basic analysis of this transporter. Other proteins will be identified by screening a cDNA library, prepared from flukes isolated from the liver of infected cows.

## Results and conclusions

Comparison of the published sequence of a previously identified ABC transporter from *Fasciola hepatica* with other transporters of this family revealed that the sequence was lacking the first six transmembrane regions of the transporter. After cloning of the missing part, it became clear that this transporter is highly homolgous not only to ABC transporters of the evolutionary close *Schistosoma mansonii*, but also to transporters of mammals. Expression of this transporter in eukaryotic cell lines should therefore allow testing the transporter’s properties, identifying substrates and blockers, and therefore getting a glance on future approaches that can be used to treat fascioliosis in animals and humans.

